# Design and optimization of candesartan loaded self-nanoemulsifying drug delivery system for improving its dissolution rate and pharmacodynamic potential

**DOI:** 10.1080/10717544.2020.1760961

**Published:** 2020-05-13

**Authors:** Ravinder Verma, Deepak Kaushik

**Affiliations:** Department of Pharmaceutical Sciences, Maharshi Dayanand University, Rohtak, Haryana, India

**Keywords:** Candesartan, D-optimal mixture design, food-effect, in vitro lipolysis, SNEDDS, pharmacodynamic study

## Abstract

During the last decades, much attention has been focused on SNEDDS approach to resolve concerns of BCS II class drugs with accentuation on upgrading the solubility and bioavailability. The present hypothesis confirms the theory that SNEDDS can reduce the impact of food on Candesartan solubilization, thereby offering the potential for improved oral delivery without co-administration with meals. The present studies describe quality-by-design-based development and characterization of Candesartan loaded SNEDDS for improving its pharmacodynamic potential. D-optimal mixture design was used for systematic optimization of SNEDDS, which showed globule size of 13.91 nm, more rapid drug release rate of >90% in 30 min and 16 s for self-emulsification. The optimized formulations were extensively evaluated, where an *in vitro* drug release study indicated up to 1.99- and 1.10-fold enhancement in dissolution rate from SNEDDS over pure drug and marketed tablet. *In vivo* pharmacodynamic investigation also showed superior antihypertensive potential of SNEDDS in normalizing serum lipid levels as compared to pure drug and marketed tablet that was executed on male Wistar rats. Overall, this paper reports successful systematic development of candesartan-loaded SNEDDS with distinctly improved biopharmaceutical performance. This research work interpreted a major role of SNEDDS for enhancing the rate of dissolution and bioavailability of poorly water soluble drugs.

## 1. Introduction

Candesartan (kan” de sar’ tan) is a BCS II class drug that is widely used alone or in combination with other agents for therapy of hypertension and heart failure. It inhibits the renin-angiotensin system by blocking the angiotensin II type 1 receptor, which prevents the vasoconstriction and volume expansion induced by circulating angiotensin II, resulting in its antihypertensive potential. It is commercially available in 4, 8, 16 and 32 mg tablets generically (Candesar/Candosa/blopress/Camperten), under the trade name Atacand. It may be brought into play to treat hypertension, isolated systolic hypertension, left ventricular hypertrophy and diabetic nephropathy. It may also be used as a second-line drug for the treatment of heart failure, systolic dysfunction, myocardial infarction and coronary artery disease. Its typical dose is 16–32 mg “quaque die” in adults which is used for the long term (Zhao & Wang, 2018).

It has the most effective antihypertensive pharmacological response. Its poor aqueous solubility results in its slow rate of dissolution and its less oral bioavailability (15%). Thus, improving its dissolution can result in improved oral bioavailability (Alshora et al., 2018).

Regardless of numerous novel inventions for delivering active pharmacotherapeutic compounds, drug administration through oral route is most desired among patients of all age groups. The acceptability of this versatile and natural oral route is attributed to ease of administration, cost-effectiveness, and improved compliance by patients (Pal et al., 2013).

SNEDDS is a novel approach in drug delivery and solves deficiency related to the delivery of BCS II class medicaments (Thomas et al., 2013; Verma et al., 2017). These are described as clear systems that consist of oils, surfactants, co-surfactant, which result in ultrafine oil/water emulsion with mean globule size distribution <100 nm upon emulsification in the gastric milieu (Gahlawat et al., 2019). They help in possessing higher solubilization capacity, leading to the addition of medicament inside the oil phase (Khalifa et al., 2019; Tong et al., 2019; Kuncahyo et al., 2019). The excipients contained in the SNEDDS tend to facilitate bioavailability of the drugs not merely by improving drug solubility and permeability but by circumventing the metabolism by liver microsomes and inhibiting P-gp efflux, along with the ability to facilitate lymphatic drug absorption. Several literature studies on various self-emulsifying formulations have reported potential improvement in the bioavailability of various drugs (Kalantari et al., 2017; Alhasani et al., 2019; Patki et al., 2019; Alskär et al., 2018).

Based on these considerations, the main aim of this research is use of quality-by-design (QbD) approach for the systematic drug product development that helps in attaining consistent quality and robust performance.

QbD approach provides product and process understanding for continuous improvement. Among diverse elements of QbD, the experimental designs are considered as a pivotal tool, which provides maximal information using minimal experimentation (Heshmati et al., 2013).

## 2. Materials and methods

### 2.1. Materials

Candesartan pure drug was a generous gift from Sun Pharmaceuticals Laboratories, Gurugram, Haryana. Capmul PG-8 and Kolliphor EL were kindly supplied by IMCD India Private Limited, Delhi. Transcutol P (Gattefosse) and Pancreatin (Loba Chemie). The marketed tablet (Candesar 16 mg batch no. 9040580) was dispensed from a community pharmacy. Other materials and reagents used in this report were of analytically research grade.

### 2.2. Methods

#### 2.2.1. Investigations of candesartan solubility in excipients

To determine and plot the possible emulsifying regions, it is necessary to elucidate the solubility of Candesartan in different oil or surfactant components (Gué et al., 2016).

The saturation solubility study of Candesartan in various vehicles was investigated. An excessive quantity of Candesartan was incorporated into each ingredient (2 g) in screw-capped glass vials. Vortex mixer (Genius, India) was used to assist the proper blending of Candesartan and vehicles (Gamal et al., 2017).

For shaking of the mixture, thermostatically controlled shaker (Calton) was used at 100 rpm for 72 h at 25 ± 0.5 °C. After removal of samples, centrifugation was done at 5000 rpm for 30 min. The supernatant was collected from the solution and a 0.45 μm membrane filter (Millipore) was used for filtration. The concentration of Candesartan was deliberately using a ultra-violet spectrophotometer (UV 1700, Shimadzu, Japan) at 254 nm. The experiment was repeated in triplicates (ElShagea et al., 2019). Self-emulsification capacity of surfactant and oil was investigated for choosing their best combination. 10 ml of each surfactant solution (10% w/w aqueous solution) was titrated with each oil (Borhade et al., 2008). Volume of oil when it converted emulsions clarity into turbid, was noted and combination was selected, which offered the highest quantity of oil emulsified (Bharti et al., 2018; Verma et al., 2018).

1:1 (Smix) was formulated with each co-surfactant for selection of co-surfactant and various formulations were developed with chosen oil and Smix. 500 mg of each formulation was blended with 500 ml of distilled water and resultant’s emulsion clarity was noted down (Lee et al., 2018).

#### 2.2.2. Construction of pseudo-ternary phase diagram

It was plotted (in the presence of medicament) with surfactant, co-surfactant and oil, and every one of them speaks to a side of the triangle. Ternary blends with shifting organizations of these three ingredients were readied, bringing about an aggregate sum of 1 g. Smix were blended in five proportions, to be specific; 1:1, 1:2, 2:1, 3:1 and 1:3. Oil and Smix proportion were blended completely in nine diverse weight proportions from 1:9 to 9:1 in various glass vials with the goal that most extreme proportions were formulated for the examining to outline the limits of phase accuracy created in this diagram (Panigrahi et al., 2019). Its outlines were created utilizing the water dilution method. The development of the nanoemulsion was outwardly seen as transparent/clear and effectively flowable/dispersible with low consistency o/w nanoemulsion and set apart on it. The measure of every part (oil, Smix) now was recorded and introduced in it (Johnson et al., 2009; FDA, 2012). It was built utilizing Chemix software (Chemix Version 4.50) (Kim et al., 2018).

#### 2.2.3. Design of experiment (DOE) to optimize SNEDDS

Recently, many statistical experimental designs have been utilized for more expertly improved plans utilizing fewer investigations, and to gauge the relative significance among other factors. Among various statistical optimization tools, D-optimal mixture design is one of the most mainstream surface approaches for optimizing SNEDDS since; it limits the difference related to the assessment of coefficients in a model and delivers the ideal subset by taking into account the criteria for boosting data grid determinants. In addition, this design considers the total system of SNEDDS as 100%, while other designs do not consider (Son et al., 2018; Mura et al., 2005).

The components were X_1_ as oil percentage (Capmul PG-8), X_2_ as surfactant percentage (Kolliphor EL), and X_3_ as a co-surfactant percentage (Transcutol P) to formulate SNEDDS with least globule size. The design of the experiment helped us both analyze and record the response (Y) as outcomes, namely globule size (Y_1_), %CDR (Y_2_) and self-emulsification time (Y_3_). Design Expert ® Software, Trial Version, was used to harmonize the regression equations further to calculate the recorded responses (Hosny et al., 2019).

##### 2.2.3.1. Determination of globule size

Zetasizer ZS nano series; Malvern Instruments, Malvern, UK based on Photo correlation scattering was used for the assessment of globule size of the nano-emulsion after 100 folds dilution of SNEDDS formulation with distilled water (Eleftheriadis et al., 2019).

##### 2.2.3.2. Dissolution testing

For this test, 900 ml of 0.35% Polysorbate 20 in 0.05 M Phosphate buffer media of pH 6.5 ± 0.05 at 37 °C ± 0.5 °C was utilized as dissolution media in USP II apparatus (Distek, USA) at 50 rpm (Pal et al., 2016). The capsule containing SNEDDS equivalent to 16 mg of Candesartan was incorporated into the buffer media after initiating rotation of the paddle. Aliquots (5 ml) were withdrawn after 30 min and analyzed by UV spectroscopy at λmax 254 nm (Patel et al., 2019). The experiment was repeated in triplicates.

##### 2.2.3.3. Emulsification time

By the reported method, a self-emulsification study was carried out on each of the mixtures. Briefly, 1 ml of optimized SNEDDS was added into 500 ml of Millipore water and agitated at approximately 100 rpm with a magnetic stirrer. Emulsion formation and dispersibility time was noted (Rangaraj et al., 2019).

#### 2.2.4. Evaluation parameters

Based on optimization results, optimized formulation was chosen to carry out characterization and further investigations such as transmittance test, cloud point, globule size and zeta potential.

##### 2.2.4.1. % transmittance test

While preparing SNEDDS formulation for the oral route, there are chances of precipitation of the medicament following dilution in lumen of the gut and for that % transmittance is measured. 1 g of SNEDDS was diluted with 100 ml Millipore water and measurement was done at λmax 254 nm using UV spectrophotometer 1700, Shimadzu, Japan and performed in triplicates using water as blank (Zhang et al., 2019).

##### 2.2.4.2. Robustness to dilution

Robustness was investigated following 100 times dilution of optimized formulation with various mediums including 0.1 N HCl, Acetate buffer (pH 4.5) and phosphate buffer (pH 6.8). After storing these samples for 24 h, they were checked for phase partitioning or precipitation of medicament (Ahsan & Verma, 2017).

##### 2.2.4.3. Viscosity measurement

Hyrdromotion viscometer (Brookfield Engineering, USA) was used for measuring the viscosity of the optimized SNEDDS formulation. This test confirms whether the nano-emulsion is o/w or w/o type. If nano-emulsion has a high viscosity, then it indicates that it is w/o type and vice-versa (Abhijit et al., 2007; Wu et al., 2015).

##### 2.2.4.4. Cloud point (TCloud) determination

Measurement was done, following 100 times dilution of 1 g optimized formulation with double distilled water and kept in a water bath for gradual increment in temperature of formulation (5 °C increments) (Shoshtari et al., 2010; Agrawal et al., 2015).

##### 2.2.4.5. Determination of drug content

For this, drug content was extracted after its 10 times dilution with methanol (v/v) and centrifugation was performed for 30 min at 10,000 rpm. Then, supernatant was diluted with methanol (2.5 folds) which was analyzed for drug content through UV spectrophotometer 1700, Shimadzu, Japan at 254 nm and performed in triplicates (Baloch et al., 2019).

##### 2.2.4.6. Measurement of globule size, polydispersity index (PDI) and zeta potential

The optimized formulation was diluted freshly at a ratio of 1:100 w/v and blended for 1 min before analysis of globule size, PDI and zeta potential measured by Zetasizer ZS nano series; Malvern Instruments, Malvern, UK. This was performed in triplicates and depicted as mean ± standard deviation (Bang et al., 2019; Alwadei et al., 2019; Enin, 2015).

##### 2.2.4.7. Multi-media dissolution testing

The SNEDDS formulations (equivalent to 16 mg of Candesartan, size “00” capsules) were dropped in dissolution medium of pH 1.2, 4.5 and 6.8 at 37˚C ± 0.5 °C in USP apparatus II (paddle) (Distek, USA). Aliquots (5 ml) were withdrawn at predetermined time points, an equal volume of fresh buffer media was incorporated after each sampling and 0.45-μm Millipore membrane filter was used for filtration. Drug release was measured using UV spectrophotometrically at 254 nm after appropriate dilution with media against equivalent proportions of excipients as blank in triplicates (Jakab et al., 2018).

##### 2.2.4.8. Comparative study of in vitro dissolution testing of optimized formulation with pure medicament and marketed formulation

In vitro dissolution testing was performed with pure Candesartan, optimized SNEDDS and marketed tablet (Candesar 16 mg batch no. 9040580) in the “USP type-II dissolution apparatus (Distek, USA) as per dissolution conditions specified by FDA guidelines”. Each formulation was kept in 0.35% Polysorbate 20 in 0.05 M Phosphate buffer media of pH 6.5 ± 0.05 at 37 °C ± 0.5 °C at 50 rpm. After 5, 10, 15, 30 and 60 min, 5 ml of aliquots were analyzed using UV spectrophotometer at 242 nm. After every sampling, fresh buffer was utilized as replacement media.

##### 2.2.4.9. Investigation of food effect by dynamic in vitro lipolysis

The literature reported that SNEDDS avoids the food effect in terms of drug discharge. For proving this theory, the dissolution of SNEDDS formulation was conducted in modified Fa/FeSSIF V-2 media to mimic in vivo milieu.

For best clinical pertinence, it is essential to lead in vitro analysis of medicament to imitate in vivo environment as intently as could reasonably be expected. This investigation is valuable for two specific rationales. Firstly, quantification of rate and degree of lipolysis by pH-stat titration, which can set up how the formulation can be influenced by equilibrium solubility and dispersion qualities of SNEDDS. Also, after the response is ended, the post lipolysis item can be examined to foresee how much content of the medicament is in solubilized or precipitated form. This model can dependably foresee the capacity of such formulations to upgrade oral assimilation of medicaments that have poor aqueous solubility.

In the present investigation, the dynamic in vitro lipolysis investigation was a rendition of the strategy recently depicted by Mohsin (2012). Each 520 mg of optimized formulation was dispersed into 36 ml of FaSSIF V-2 and FeSSIF V-2 whose composition is shown in Table 1 (Mosgaard et al., 2015; Xiao et al., 2016; Sassene et al., 2014). Concentrations of Ca^++^, bile, phospholipid (PL), and sodium chloride (NaCl) were preferred to imitate typical concentrations occurring in FaSSIF V-2/FeSSIF V-2. During the early phase of dispersion, 6.5/5.8 ± 0.05 of pH was adjusted with NaOH or HCl.

**Table 1.** Composition of biorelevant media used during *in vitro* lipolysis.

Chemical	FaSSIF V-2	FeSSIF V-2
Sodium taurocholate (mM)	3	10
Glyceryl monooleate (mM)	–	2
Sodium oleate (mM)	–	5
Lecithin (mM)	0.2	0.8
Maleic acid (mM)	19.12	55.02
NaCl (mM)	68.62	125.5
NaOH (mM)	34.8	81.65
pH	6.5	5.8

FaSSIF V2: fasted state simulated intestinal fluid V-2; FeSSIF V-2: fed state simulated intestinal fluid V2.

The stirring process was utilized for the emulsification of SNEDDS formulations on a magnetic stirrer with a hot plate at 37 °C, earlier to the incorporation of enzyme. 4 ml of pancreatic extract [formulated by suspending pancreatin powder (1 g) in digestion buffer (5 ml) and vortex blending for 15 min. Ultracentrifugation was performed and supernatant of pH 6.5/5.8 containing 800 TBU/ml of pancreatic lipase/co-lipase] addition initiates lipolysis which was continuous for next 30 min with a pH-stat titration unit (Metrohm, Switzerland), which was maintained a constant pH of 6.8/5.8. During lipolysis, production of fatty acids (FAs) results in an elevation in pH of biorelevant media, 0.2 M NaOH solution was utilized for maintaining pH. The progress of drug release in digestion buffers was monitored directly by UV analysis at 254 nm (Williams et al., 2012; Alshamsan et al., 2018). By following this protocol, the present strategy was seen as robust and estimated values were reproducible.

##### 2.2.4.10. Stability studies

30 capsules containing optimized Candesartan SNEDDS (each capsule contains Candesartan SNEDDS equivalent to Candesartan 16 mg) were packed in 60 cc HDPE Bottle and were placed in stability chamber (Thermolab, India) at 40 ± 2 °C/75 ± 5% RH for 6 months after sealing bottles. After 1 M/3M/6M, samples were removed and evaluated in terms of description, drug release and disintegration time of formulation (Izham et al., 2019).

##### 2.2.4.11. Pharmacodynamic studies

Candesartan has a dose-dependent pharmacodynamic effect and that’s why comparative in vivo study was investigated with the marketed tablet dosage form.

The ethical permission for the pharmacodynamic study of Candesartan SNEDDS formulation in rats was granted by the “Institutional Animal Ethical Committee (IAEC), Maharshi Dayanand University, India (Reg. no. 1767/RE/S/14/CPCSEA, vide reference no. 153-165 dated 14/12/2018). Male Wistar rats having weight 150–200 g were purchased from Lala Lajpat Rai University of Veterinary and Animal Sciences, Hisar. Animals were maintained as per the guidelines of CPCSEA, India”. All animals were kept in plastic cages; six animals per cage were provided accommodation with 12 h of light/dark cycle, at 25 ± 2˚C, with pelleted food, tapwater and libitum were fed.

All animals were adapted to research facility environment for 1 week prior to experimentation and fasted for 12 h before the experiment, they were made available with libitum access to water. This investigation in rats was carried out according to the method as depicted in previous literature (Kumar & Nanda, 2018) with a few modifications. The animals were separated into five groups (total 30 rats; each group having 6 rats), i.e., “control treatment group (CTG), placebo treatment group (PTG), reference treatment group (RTG), test (TTG) and marketed treatment group (MTG)”.

The effect of Candesartan loaded SNEDDS (TTG) on lipid profile was determined by comparison with Candesartan drug (RTG) and SNEDDS without Candesartan (PTG). Marketed, test, reference and placebo formulation was diluted with 2.0% acacia solution. Each treatment group received 18% NaCl solution as a dose of 10 ml/kg/day bodyweight daily for 4 weeks (Mao et al., 2015). TTGs, RTGs, MTGs, and PTGs additionally receive test formulation, reference formulation, marketed and placebo formulation, respectively, for 4 weeks. The administered oral dose of the test product and reference product was equivalent to 0.3 mg/kg/day of Candesartan (Gleiter et al., 2006).

Alteration in MSBP was measured (0 days and after 28 days) with noninvasive blood pressure (NIBP) (AD Instruments, Australia) by using the tail-cuff method for each treatment group. One-way analysis of variance (ANOVA) with the Dunnet test was implemented to evaluate the differences in the mean of different groups using the Graph Pad version 5.0 statistical analysis software. Data are shown in mean ± standard deviation. The statistical significance level was acceptable at *p* < 0.05.

## 3. Results and discussion

### 3.1. Solubility study

**Figure 1.**
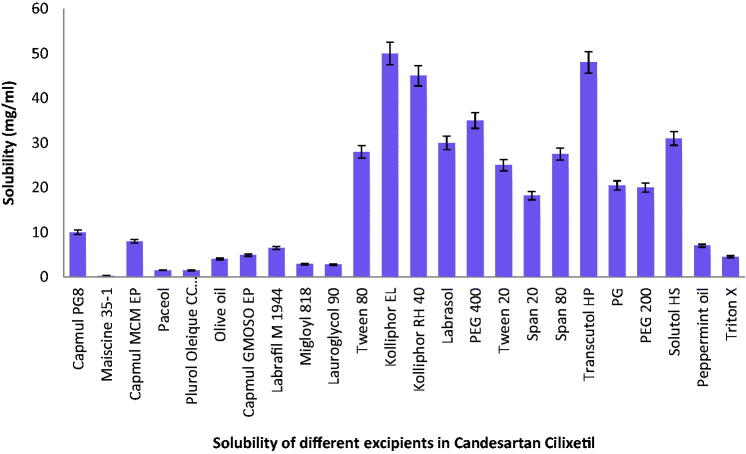
Solubility of Candesartan in various oils.

For the determination of stability of the formulation, solubility of medicament in ingredients plays a significant function because many formulations undergo precipitation before experiencing in situ solubilization. High drug solubilization is very significant for increasing the efficiency of drug loading into carriers with concomitant improvement in oral bioavailability (Parmar et al., 2011). In addition, for the development of an effective Candesartan SNEDDS, its prescribed amount should be miscible in its selected excipients with the least amount of the mixture (Qi et al., 2011). The results of Candesartan solubility in various ingredients are shown in Figure 1.

The self-emulsification feature depends on the selection of a suitable combination of ingredients. This study showed that Kolliphore EL with the highest quantity of Capmul PG-8 had been emulsified as shown in Table 2. That’s why; Kolliphore EL and Capmul PG-8 combination was selected.

**Table 2.** Emulsification of oils with different surfactant.

Surfactant (10% solution)	Oils	Volume of oil emulsified (mL)
Kolliphor EL	Capmul PG-8	0.70
Kolliphor RH 40	Capmul PG-8	0.50
Kolliphor EL	Capmul MCM EP	0.40
Kolliphor RH 40	Capmul MCM EP	0.60

Transcutol P is selected as a co-surfactant because it showed greater nanoemulsion region as compare to PEG 400 as shown in Table 3.

**Table 3.** Identification of nanoemulsion region (transparent) based on visual observation with different co-surfactants.

%Oil	%Smix	Nanoemulsion region	
		Smix Kolliphore EL : Transcutol P (1:1)	Smix Kolliphor EL:PEG 400 (1:1)
10	90	Transparent	Transparent
20	80	Transparent	Transparent
30	70	Transparent	Transparent
40	60	Transparent	Turbid
50	50	Transparent	Turbid
60	40	Turbid	Turbid
70	30	Turbid	Turbid
80	20	Turbid	Turbid
90	10	Turbid	Turbid

Turbid: nonnanoemulsion region.

Transparent: nanoemulsion region.

The surfactant creates a layer around oil droplets and diminishes surface tension between aqueous and oil phase. Additional, elevation of the concentration of surfactant results in enhancement of the spontaneity of self-emulsification. Elevation in co-surfactant concentration diminishes the area for the formation of emulsion, but it has minimal impact on dropping interfacial tension (Nepal et al., 2010). A higher value of HLB is necessary for creating o/w type emulsion. Co-surfactant is used in the Candesartan preparation mainly to minimize the surfactant ratio in the formulation (Zhao et al., 2010). Transcutol P was incorporated in the formulation to increase the solubilization of the model lipophilic drug compounds.

### 3.2. Pseudo-ternary phase diagram

**Figure 2.**
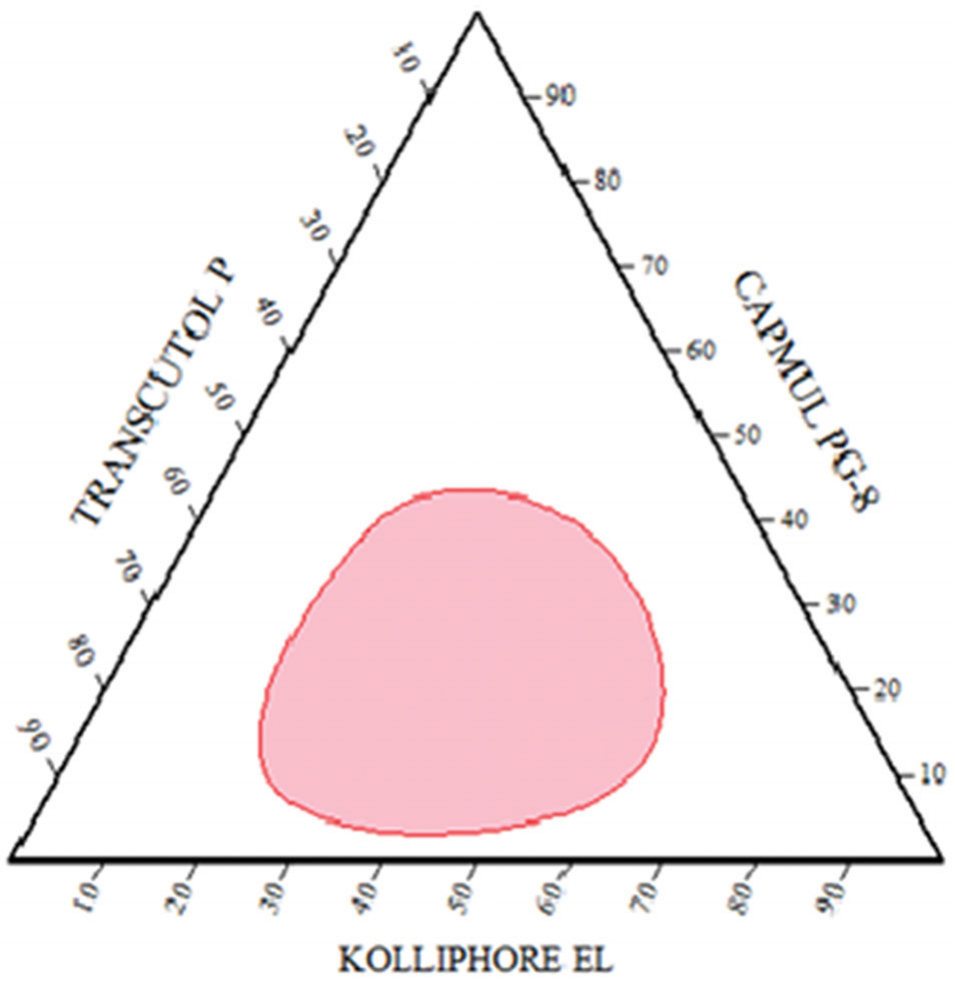
Pseudo ternary phase diagram.

It was plotted in the presence of Candesartan to recognize the self-nanoemulsifying region and for the selection of an appropriate concentration of ingredients for the development of SNEDDS. It plays a significant function to study phase behavior of formed nanoemulsions (Balakumar et al., 2013). It was constructed by using water dilution method with different amount of oil (5-90%), Smix (1:1, 1:2, 2:1, 3:1 and 1:3) and transparency for the formation of nanoemulsion as shown in Table 4. Resulted data was used for the construction of a ternary phase diagram where each vertex represents 100% of that specific ingredient. In Figure 2, the shaded area presented transparent and low viscosity nanoemulsion area in it.

**Table 4.** Result of water dilution method of Oil + Smix with Candesartan.

S. no.	% Oil	% Surfactant	% Co-surfactant	Observation
		Smix ratio 1:1		
1.	5	55	55	Transparent
2.	10	45	45	Transparent
3.	20	40	40	Transparent
4.	30	35	35	Transparent/bluish
5.	40	30	30	Transparent/bluish
6.	50	25	25	Turbid
7.	60	20	20	Turbid
8.	70	15	15	Turbid
9.	80	10	10	Turbid
10.	90	5	5	Turbid
		Smix ratio 2:1		
1.	5	63.34	31.66	Turbid
2.	10	60	30	Transparent
3.	20	53.30	26.70	Transparent
4.	30	46.70	23.30	Transparent/bluish
5.	40	40	20	Transparent/bluish
6.	50	33.30	16.70	Turbid
7.	60	26.70	13.30	Turbid
8.	70	20	10	Turbid
9.	80	13.30	6.70	Turbid
10.	90	6.70	3.30	Turbid
		Smix ratio 1:2		
1.	5	31.66	63.34	Transparent
2.	10	30	60	Transparent
3.	20	26.70	53.30	Transparent
4.	30	23.30	46.70	Transparent/bluish
5.	40	20	40	Transparent/bluish
6.	50	16.70	33.30	Turbid
7.	60	13.30	26.70	Turbid
8.	70	10	20	Turbid
9.	80	6.70	13.30	Turbid
10.	90	3.30	6.70	Turbid
		Smix ratio 3:1		
1.	5	71.25	23.75	Turbid
2.	10	67.5	22.5	Turbid
3.	20	60	20	Transparent/bluish
4.	30	52.5	17.5	Turbid
5.	40	45	15	Turbid
6.	50	37.5	12.5	Turbid
7.	60	30	10	Turbid
8.	70	22.5	7.5	Turbid
9.	80	15	5	Turbid
10.	90	7.5	2.5	Turbid
		Smix ratio 1:3		
1.	5	23.75	71.25	Turbid
2.	10	22.5	67.5	Transparent
3.	20	20	60	Transparent/bluish
4.	30	17.5	52.5	Turbid
5.	40	15	45	Turbid
6.	50	12.5	37.5	Turbid
7.	60	10	30	Turbid
8.	70	7.5	22.5	Turbid
9.	80	5	15	Turbid
10.	90	2.5	7.5	Turbid

### 3.3. Mixture design tool in the optimization and statistical analysis

For optimization Candesartan-loaded SNEDDS composition, a mixture design was used using Design Expert® software Trial Version. As shown in Table 5, fourteen experimental runs were found according to this design with two center points. Y_1_ ranged from 11.39 to 119.8 nm, Y_2_ from 86 to 98.5% and Y_3_ ranged from 15 to 41 s. The effect of different proportions of components on globule size, drug release and self-emulsification could be explained by the following equations:

**Table 5.** Composition of various SMEDDS formulation suggested by Design Expert^®^ in the study and response.

Formulation code	Excipients ratio			Y_1_ (nm)	Y_2_ (%)	Y_3_ (s)
	X_1_ (%)	X_2_ (%)	X_3_ (%)			
F1	0.10	0.40	0.50	13.68	93.2	26
F2	0.16	0.25	0.59	60.94	95	41
F3	0.17	0.40	0.43	52.23	91	25
F4	0.05	0.48	0.47	11.39	90	15
F5	0.10	0.25	0.65	22.69	97	39
F6	0.05	0.38	0.57	15.89	94.5	17
F7	0.25	0.5	0.25	87.39	86	24
F8	0.18	0.30	0.52	40.79	94.9	32
F9	0.23	0.25	0.52	119.8	91.7	36
F10	0.25	0.35	0.40	111.1	91	28
F11	0.05	0.32	0.63	13.91	98.5	18
F12	0.21	0.44	0.35	105	87.7	21
F13	0.10	0.40	0.50	13.6	93	26
F14	0.14	0.5	0.36	14.45	88	20

Independent variable: X_1_ as oil percentage (Capmul MCM EP), X_2_ as surfactant percentage (Tween 20), and X_3_ as a co-surfactant percentage (Transcutol P). Dependent variables: globule size (Y_1_), %CDR (Y_2_) and self-emulsification time (Y_3_).

The equation of the fitted model for

Globular size:

(1) $$\begin{array}{l} -14020.41{\text{X}}_{\text{1}}-6653.65{\text{X}}_{\text{2}}+1702.47{\text{X}}_{\text{3}}+37529.12{\text{X}}_{\text{1}}{\text{X}}_{\text{2}}+28622.61{\text{X}}_{\text{1}}{\text{X}}_{\text{3}}+10144.95{\text{X}}_{\text{2}}{\text{X}}_{\text{3}}\\ -55716.48{\text{ X}}_{\text{1}}{\text{X}}_{\text{2}}{\text{X}}_{\text{3}}-1162.60{\text{X}}_{\text{1}}{\text{X}}_{\text{2}}\left({\text{X}}_{\text{1}}-{\text{X}}_{\text{2}}\right)+17417.69{\text{X}}_{\text{1}}{\text{X}}_{\text{3}}\left({\text{X}}_{\text{1}}{\text{X}}_{\text{3}}\right)+14448.92{\text{X}}_{\text{2}}{\text{X}}_{\text{3}}\left({\text{X}}_{\text{2}}-{\text{X}}_{\text{3}}\right) \end{array}$$

%CDR:

(2) $$\begin{array}{c} +606.{12X}_{1^{-}}+356.{52X}_{2}+20.{23X}_{3}-1352.{14X}_{1}X_{2}-1151.{62X}_{1}X_{3}-400.{93X}_{2}X_{3}+2375.{57X}_{1}X_{2}X_{3}\\ +347.{21X}_{1}X_{2}(X_{1}-X_{2}-747.30X_{1}X_{3}(X_{1}-X_{3}-675.{56X}_{2}X_{3}(X_{2}-X_{3})) \end{array}$$

Self-emulsification time:

(3) $$\begin{array}{c} +4910.{49X}_{1}-237.{62X}_{2}+35.{43X}_{3}-7561.{89X}_{1}X_{2}-8606.{26X}_{1}X_{3}+373.{13X}_{2}X_{3}\\ +6615.{36X}_{1}X_{2}X_{3}–3791.{54X}_{1}X_{2}(X_{1}-X_{2}-5218.{51X}_{1}X_{3}\left(X_{1}-X_{3}\right)-+563.{77X}_{2}X_{3}\left(X_{2}-X_{3}\right)\\ +4910.{49X}_{1}-237.{62X}_{2}+35.{43X}_{3}-7561.{89X}_{1}X_{2}-8606.{26X}_{1}X_{3}+373.{13X}_{2}X_{3}\\ +6615.{36X}_{1}X_{2}X_{3}–3791.{54X}_{1}X_{2}(X_{1}-X_{2}-5218.{51X}_{1}X_{3}\left(X_{1}-X_{3}\right)-+563.{77X}_{2}X_{3}\left(X_{2}-X_{3})\right) \end{array}$$

Where

X_1_ = Conc. of Capmul PG-8 (Oil)
X_2_ = Conc. of Kolliphor EL (Surfactant)
X_3_ = Conc. of Transcutol P (Co-surfactant)

**Figure 3.**
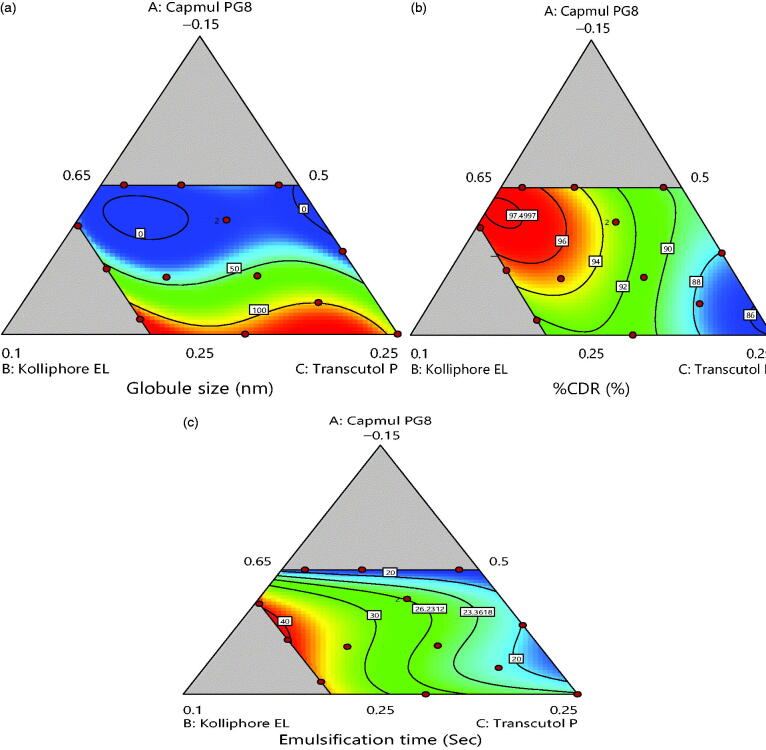
2D counter plot for (a) globule size, (b) % CDR and (c) self-emulsification time.

**Figure 4.**
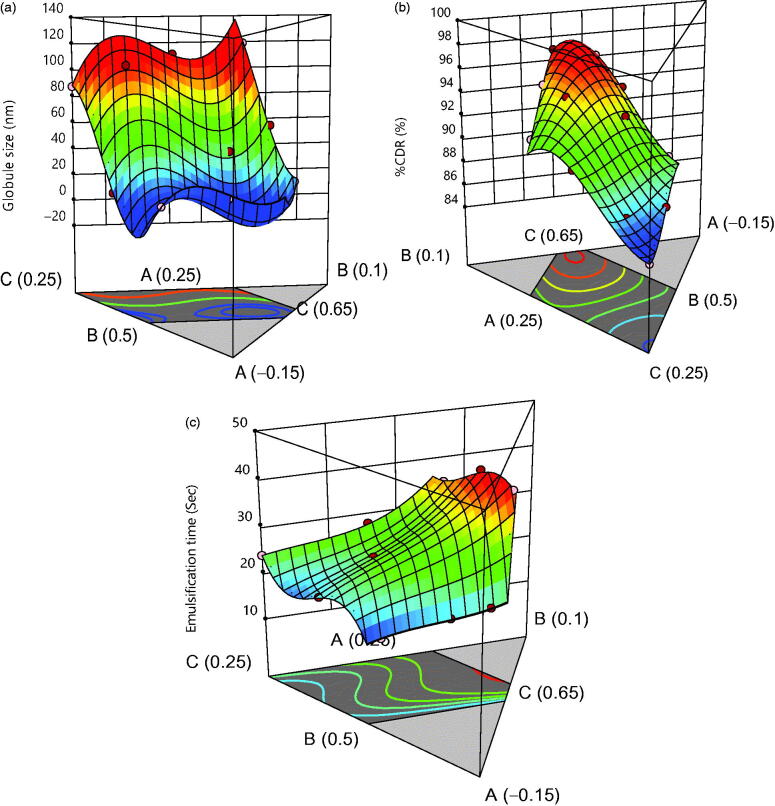
3D response plot for (a) globule size, (b) % CDR and (c) self-emulsification time.

**Figure 5.**
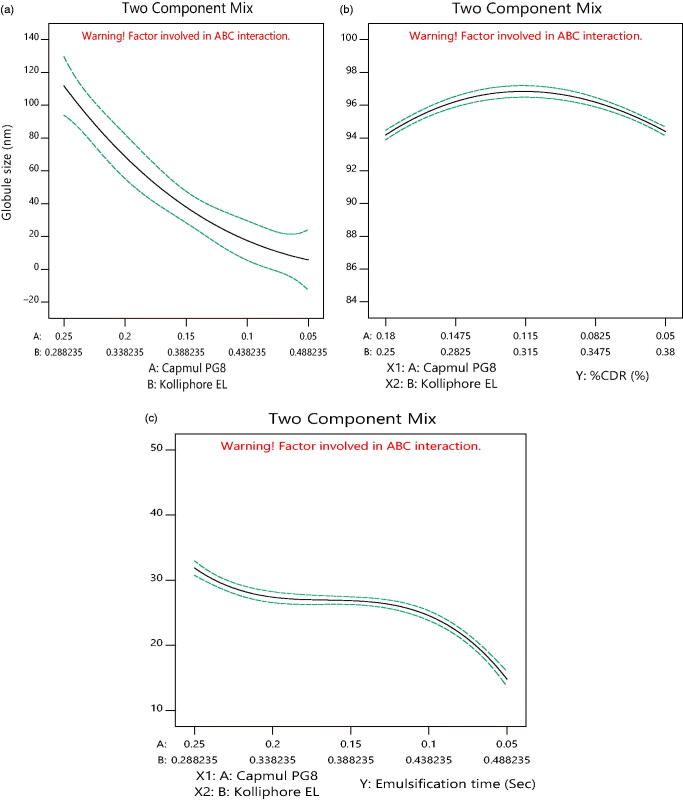
Prediction profiler (a) globule size, (b) % CDR and (c) self-emulsification time.

2-D contour plots and 3-D response plots are depicted in Figures 3 and 4 which explains the effects of X_1_, X_2_ and X_3_ on variables Y_1,_ Y_2_ and Y_3_ responses. It was observed that increment in the concentration of oil results into increment in globule size and decline in drug discharge rate and self-emulsification time also increases. But, an increase in concentration of surfactant resulted in decrease of globule size, increment in drug release rate and decrease in self-emulsification time. While Figure 5 shows the actual versus predicted graph for responses that summarized that actual and predicted responses are approximately very close. Within the triangle image, the area other than gray indicates minimum globule size area, maximum %CDR and minimum self-emulsification time.

**Figure 6.**
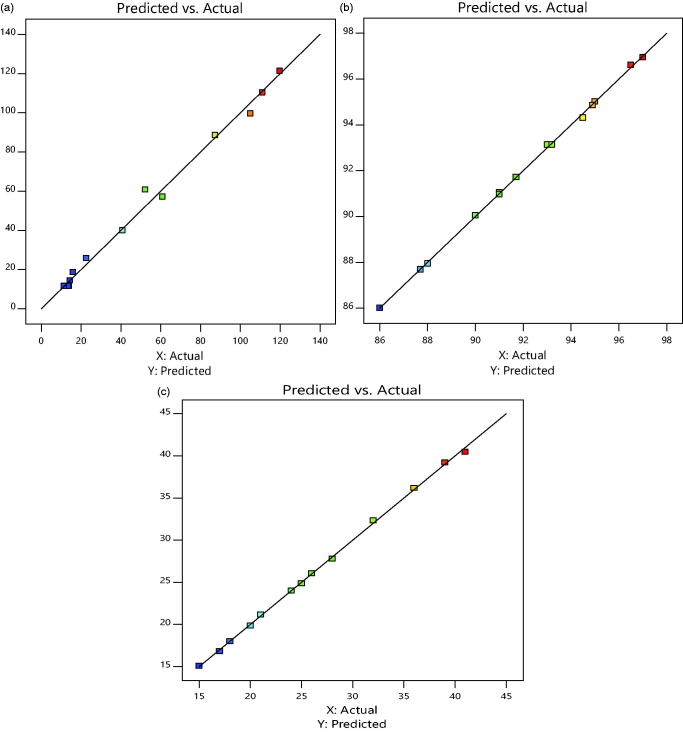
Actual versus predicted graph for response: (a) globule size, (b) % CDR and (c) self-emulsification time.

Both 2-D, 3-D contours and Equations (1, 2, 3) indicated high ratios of oil that had significantly decreased the globule size, while surfactant and co-surfactant increased it up to a limit in the formulation. The same occurs in response Y_3_ and Y_2_ up to a limit, and then it starts to increase as shown by the prediction profiler in Figure 6. Equations 1, 2, and 3 of regression helped formulate the optimized formulation. The results of ANOVA are depicted in Table 6.

**Table 6.** Result of ANOVA.

Result of ANOVA						
Response	Sum of squares	df	Mean square	*F* value	*p* Value	Model
Y_1_	21674.05	9	2408.23	62.90	0.0006	Significant
Y_2_	147.68	9	16.41	767.34	<0.0001	Significant
Y_3_	864.26	9	96.03	648.47	<0.0001	Significant

**Figure 7.**
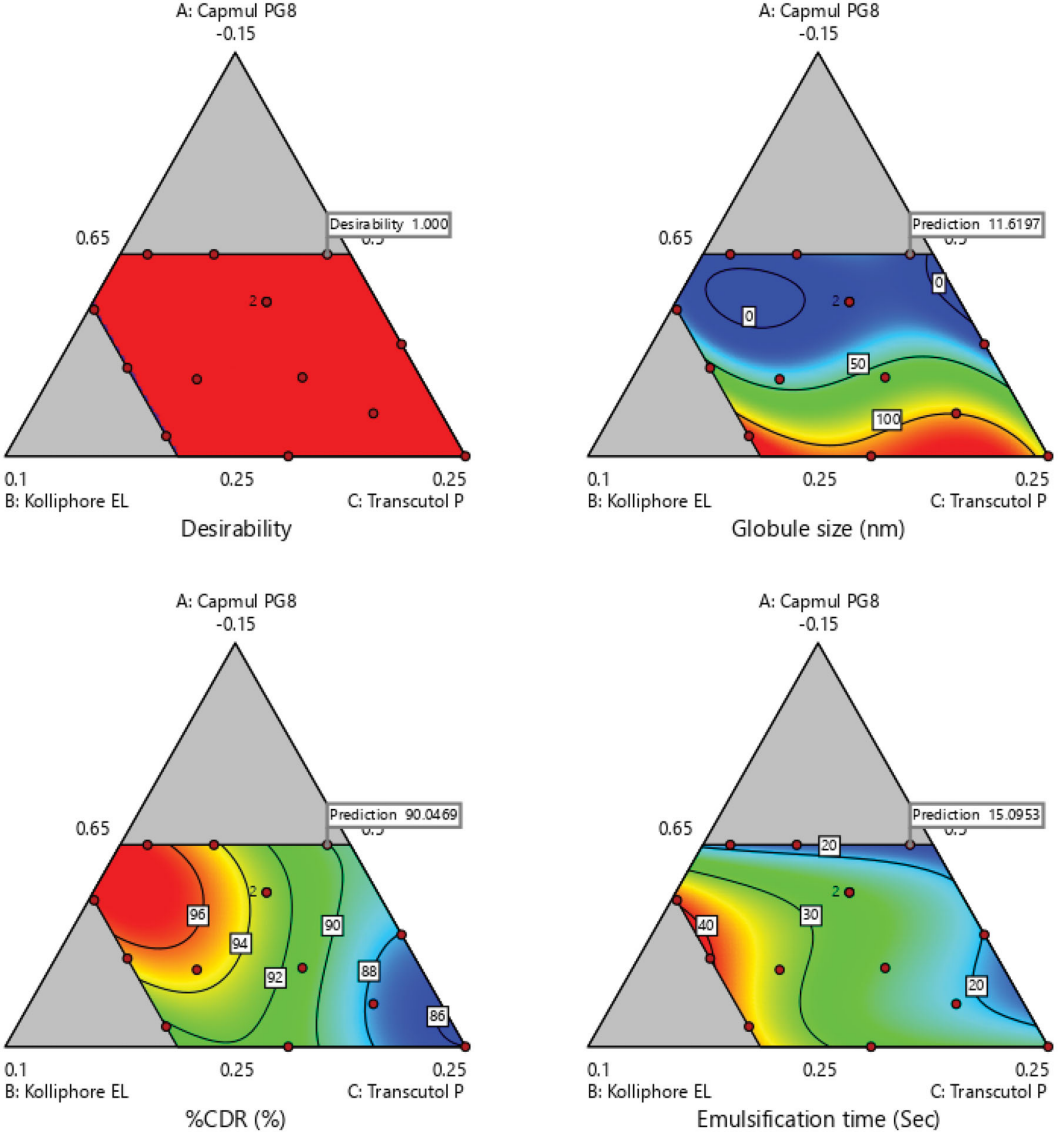
Desirability index for optimization of formulation.

The combined application of RSM and the desirability approach results into a more powerful method for finding an optimal balance between the responses. This combination has resulted in a new method called “Desirability Optimization Methodology or DOM” (Derringer, 1994). Desirability index is used for factor optimization in multi-response system that is based on the transformation of all the obtained responses from different scales into a scale-free value (Amdoun et al., 2018). The values of desirability functions lie between 0 and 1. The value 1 corresponds to the optimal performance for the investigating factors, while the value 0 is attributed when the factors result an undesirable response. The desirability index of the formulation was 1 which confirmed that the investigating factors resulted in the optimal performance of the formulation as shown in Figure 7 (Jeong & Kim, 2009).

The optimized formulation of Candesrtan loaded SNEDDS consist of 5% Capmul PG-8, 32% Kolliphor EL and 63% Transcutol P with globule size of 13.91 nm, 98.5% drug release within 30 min and 18 s self-emulsification time with desirability index value of 1.

### 3.4. Evaluation parameters

#### 3.4.1. % Transmittance

It was determined to evaluate the stability of the optimized nanoemulsion of SNEDDS. It also gave a proposal about the features of formulation such as size and uniformity of the globules. It was found to be 99.98 ± 0.5% which confirmed its clarity after dispersion into buffer media. Also, it confirmed that there are no chances of drug precipitation and optimized formulation had good solubilization capacity after dispersion.

#### 3.4.2. Robustness to dilution

The generation of uniform nano-emulsion from SNEDDS is very significant in various mediums as medicaments may precipitate out in vivo which may have an impact on the assimilation of medicaments. Optimized formulation was exposed to various media after 100 times dilution to mimic the in vivo conditions. Even after 24 h, the optimized formulation did not show any signs precipitation, haziness or separation of phase which made certain the stability of formulation. These outcomes ensured the prospect of a uniform profile of drug discharge during in vivo conditions.

#### 3.4.3. Viscosity

Viscosity of optimized formulation was found to be 168 ± 5 cps which was measured by Brookfield hydromotion viscometer in triplicates. This confirmed that this formulation can be easily transferred to any container or a capsule shell for its storage.

#### 3.4.4. Cloud point (TCloud) determination

It helps in examining the impact of temperature on the phase behavior of formulation which is one of the serious issues related to nanoemulsions, particularly when using nonionic surfactants. “It is the temperature above which the formulation transparency turns into cloudiness. An ideal formulation should remain as a single-phase clear system at its storage temperature and the temperature of its proposed use.” At high temperature, phase separation can arise because of the decline solubility of the surfactant in aqueous. It can decline both drug solubilization and formulation stability that’s why the cloud point should be over 37 °C of the formulation (Verma et al., 2017). The cloud point for the optimized SNEDDS formulation was much higher (70 °C) which shows that this formulation is stable at physiological temperature.

#### 3.4.5. Drug content

Drug content was measured by VU spectrophotometrically in optimized SNEDDS formulation which was found to be 100.05 ± 1.2% which confirms the accuracy of dose in the formulation.

#### 3.4.6. Globule size, PDI and zeta potential

**Figure 8.**
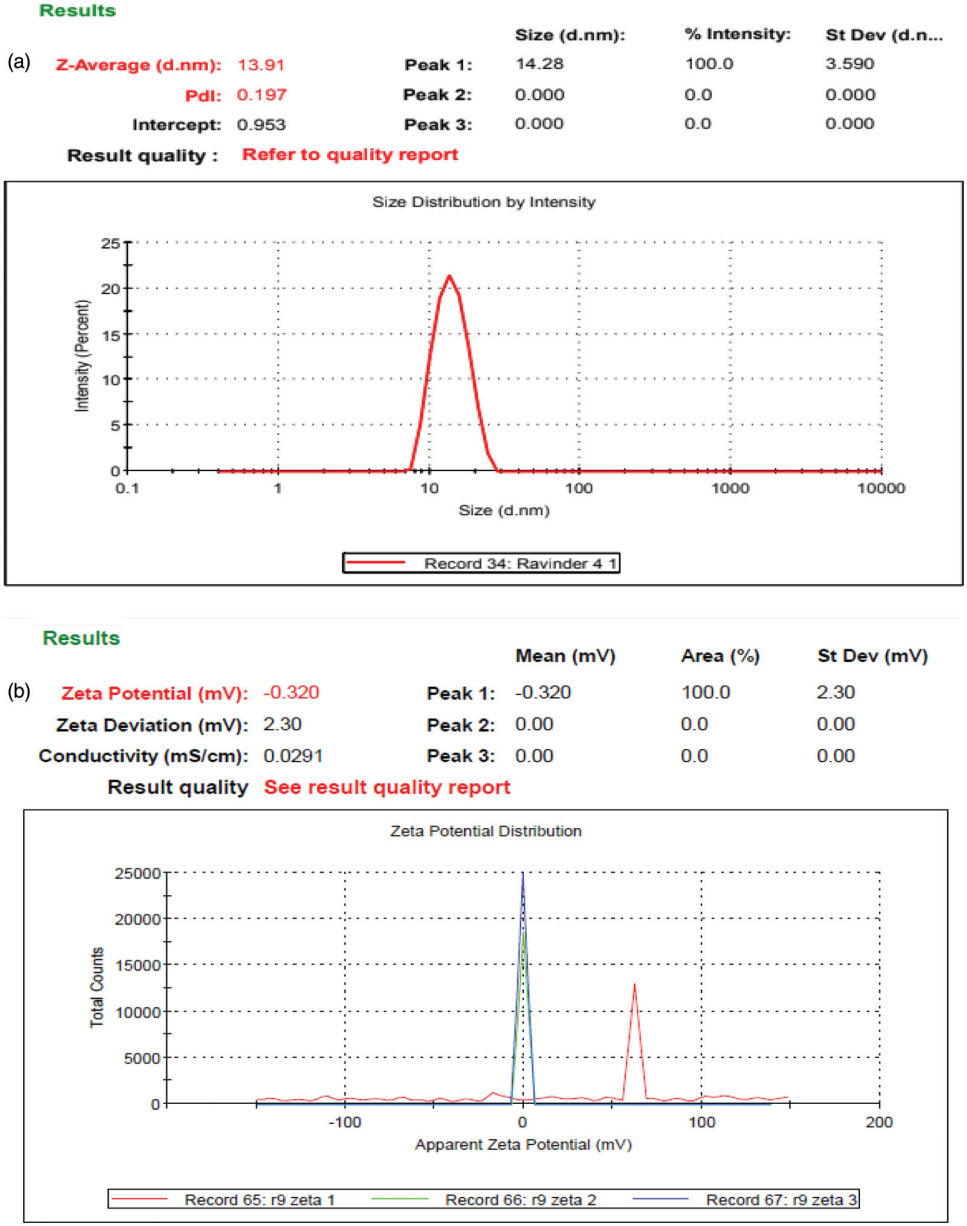
(a) Globule size and PDI. (b) Zeta potential of optimized formulation.

Globule size is of the mainly significant qualities of nanoemulsion for stability assessment and a basic advance in the pathway of improving assimilation of medicament. Its smaller size results in greater interfacial surface area for assimilation of medicament and enhanced bioavailability. Hence, its smaller size may govern the effective discharge of medicament (Eltobshi et al., 2018). The globule size of optimized formulations specifies that droplets of emulsion are in nanometric range (13.91 nm) with a PDI value less than 0.5 which indicates uniformity in the globule size distribution and zeta potential value of –0.32 mV as shown in Figure 8.

The stability of colloidal dispersions depends on the value of zeta potential which one is its significance. For smaller globules, high zeta potential will confirm electrically stability because increment in surface charge opposes the aggregation of particles. When the potential is high, repulsion exceeds attraction and the dispersion will not be deflocculated or break. In the present study, the zeta potential of optimized formulations was negatively charged due to the presence of nonionic surfactants that create a -vely charged interface at neutral pH (Choi et al., 2014, Shakeel et al., 2013).

#### 3.4.7. Multi-media dissolution testing

**Figure 9.**
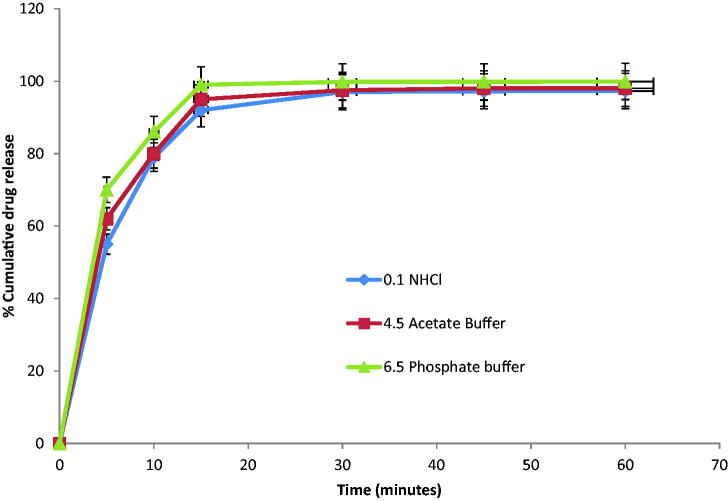
Multi-media dissolution testing of Candesartan loaded SNEDDS (*n* = 3).

The in vitro dissolution profile of optimized formulation was investigated in various dissolution media whose results are shown in Figure 9 and Table 7 (Zhang et al., 2010).

**Table 7.** Multi-media dissolution testing of Candesartan loaded SNEDDS (*n* = 3).

Time (min)	0.1 N HCl	4.5 acetate buffer	6.5 phosphate buffer
0	0	0	0
5	55 ± 2.3	62 ± 1.2	70 ± 1.1
10	79 ± 1.4	80 ± 1.7	86 ± 0.5
15	92 ± 2.1	95 ± 1.9	99 ± 1.2
30	97 ± 1.3	97.5 ± 0.7	99.8 ± 1.8
45	97.2 ± 0.9	98 ± 2.5	99.8 ± 2.3
60	97.3 ± 1.0	98 ± 2.8	99.9 ± 2.5

Data are presented as the mean ± SD.

It was concluded that drug discharge reached over 80% in 15 min in all media. Though, a mild decline or fluctuation in drug release was found at pH 1.2 and 4.5. Overall, the optimized formulation resulted in extremely improved drug release in multi-media dissolution testing.

#### 3.4.8. Comparative dissolution testing of optimized formulation with marketed tablet and pure drug

**Figure 10.**
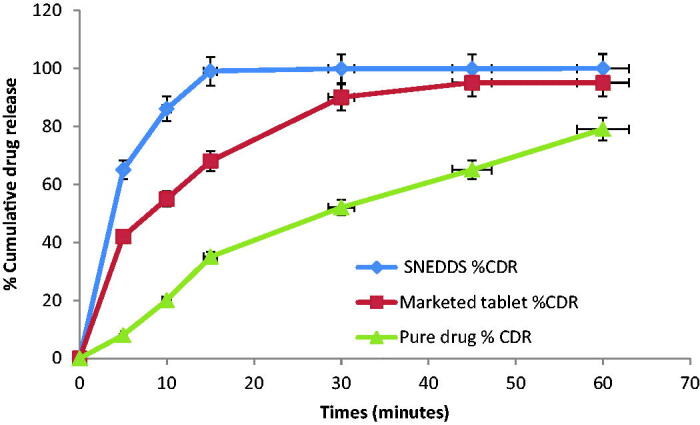
Comparative dissolution study of Candesartan loaded SNEDDS, marketed tablet and pure drug (*n* = 3).

This study was conducted with optimized formulation, marketed tablet and pure drug. It was summarized that the rate of drug discharge for the optimized formulation is more than the marketed tablet and pure drug from the results as summarized in Figure 10 and Table 8.

**Table 8.** Comparative dissolution study of Candesartan loaded SNEDDS, marketed tablet and pure drug (*n* = 3).

Time (min)	SNEDDS (%)	Marketed tablet (%)	Pure drug (%)
0	0	0	0
5	70 ± 1.1	42 ± 2.1	8 ± 2.4
10	86 ± 0.5	55 ± 1.9	20 ± 1.5
15	99 ± 1.2	68 ± 2.5	35 ± 1.7
30	99.8 ± 1.8	90 ± 2.7	50 ± 1.8
45	99.8 ± 2.3	95 ± 2.9	51 ± 2.7
60	99.9 ± 2.5	95 ± 1.0	52 ± 2.0

Data are presented as the mean ± SD.

This analysis showed up to 1.99 and 1.10-folds improvement in dissolution rate from optimized SNEDDS over pure drug and marketed tablet. These outcomes resemble with data which is obtained by several other researchers. This enhanced dissolution was likely ascribed to the accompanying basis. First, the crystalline structure of API alters into an amorphous state in which one is thermodynamically stable and offers solid-to-liquid phase transition effortlessly in SNEDDS. It is well established that this conversion of form enhances its rate of dissolution owing to elevated disorder and high energy form of the amorphous state (Liu et al., 2010, Verma & Kaushik, 2019, Kassem et al., 2016). Another reason is due to the existence of drugs as solubilized molecules inside nanoemulsion globules and nanosized suspended drug particles forming SNEDDS. All the samples showed faster drug release than unprocessed raw Candesartan because this aerophilization had relatively fewer effects on the dissolution than the creation of a high energy amorphous phase, decline in particle size and decline of surface tension of the dissolution medium.

#### 3.4.9. Assessment of food effect with dynamic in vitro lipolysis

One of the most mind-boggling and inadequately comprehended parts of SNEDDS is that they interact with GI content which has a direct impact on their performance. Digestion of dietary TG in the small intestine (SI) is generally extremely quick and various nonionic esters act as substrates of pancreatic lipase or other esterases. This process may aid the dispersion of the medicament in the existence of BS/PLs from SNEDDS and advances its retention. Therefore, lipid digestion examination can be vital because they forecast the chance of precipitation of the formulation and medicament in the intestinal lumen.

Alterations in solubilization capacity that arises throughout this process were of great significance to evaluate food effects through in vitro lipolysis (Alshamsan et al., 2018). During this investigation, it was vital to examine if there was any chance of precipitation of medicament or loss of medicament arising within 30 min. The results of the fasting state confirmed that Candesartan was present in solubilized form in the optimized formulation which leads to approximately 97.2 ± 0.7% drug discharge. While similar outcomes were obtained under fed state where the drug discharge was estimated to be 96.33 ± 0.9% which suggested that optimized formulation was able to keep Candesartan in solubilized form which is crucial for assimilation of drug. So, SNEDDS avoids the food effect in terms of drug discharge which has been reported in the literature was found to be a true hypothesis in the case of SNEDDS formulation. This suggests that SNEDDS overcome the influence of food on drug discharge. Thus, SNEDDS would enhance patient compliance, specifically in patients who are not able to take their medicines with food.

During lipolysis, the continuous digestion of the SNEDDS and generation of digestion products leads to a decline in the solubilization capacity and precipitation of Candesartan. Since, the lipid to drug ratio is higher for the SNEDDS that results into its higher the solubilizing capacity.

#### 3.4.10. Stability study

The optimized formulation was physically stable in terms of description/drug release/disintegration time. Stability data for Candesartan SNEDDS formulation has been given in Table 9. From stability data, it was observed that there are no significant differences in physicochemical parameters of Candesartan SNEDDS formulation from initial to 6 M accelerated stability condition (40 °C/75%RH) and hence, it was concluded that Candesartan SNEDDS formulation is stable.

**Table 9.** Stability data of Candesartan SNEDDS formulation (*n* = 6).

Test parameter	Initial	40 °C/75% RH/1M	40 °C/75% RH/3M	40 °C/75% RH/6M
Description	Whitish colored capsules containing clear liquid	Whitish colored capsules containing clear liquid	Whitish colored capsules containing clear liquid	Whitish colored capsules containing clear liquid
% CDR	99.9 ± 2.5	98.8 ± 2.8	98.0 ± 2.7	97.5 ± 2.0
Disintegration time	5 ± 0.5 min	5 ± 0.5 min	6 ± 1 min	6 ± 1.0 min

%CDR: percentage of cumulative drug release. Data are presented as the mean ± SD.

#### 3.4.11. Pharmacodynamic study

Pharmacodynamic study was carried out with optimized Candesartan loaded SNEDDS formulation (F11). Pharmacodynamics study results for MSBP (Mean systolic blood pressure) for each group have been given in Table 10 that were collected by NIBP (AD Instruments, Australia). The one-way ANOVA with Dunnet analysis showed a significant difference in the percentages of parameters between the positive CTG, PTG, MTG, RTG and Test treatment group (*p*  < 0.05).

**Table 10.** MSBP profile in experimental animals with mean ± std. deviation (*n* = 6).

Parameter	0 Day	Control	Placebo	SNEDDS	Marketed Tablet	Pure drug
MSBP	119.7 ± 3.39	119.5 ± 3.62	164.7 ± 3.78***	126.8 ± 2.14**	133.5 ± 2.43***	143.8 ± 2.79***

MSBP: mean systolic blood pressure. Data are presented as the mean ± SD.

***p < 0.001, **p < 0.01, and *p < 0.05 (*as compared to control).

*p* < 0.001 (highly significant), *p* < 0.01 (significant), and *p* < 0.05 (less significant).

**Figure 11.**
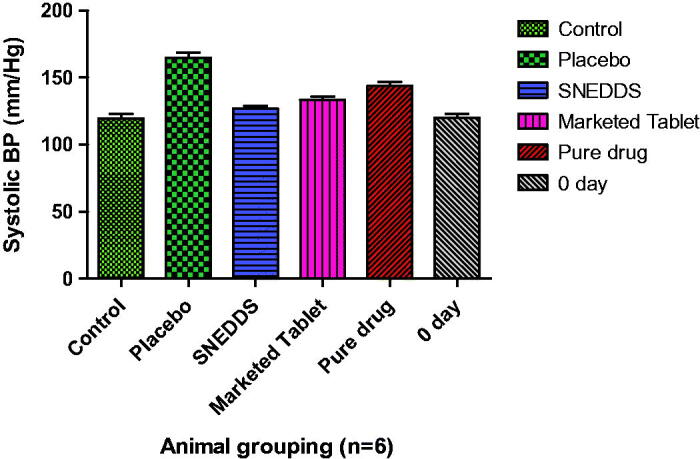
Comparative systolic blood pressure of each treatment group (*n* = 6).

Following the administration of a high-fat diet for 28 days, all the animal groups revealed a considerable rise in the systolic blood pressure levels signifying the hypertension. Treatment with TTG showed remarkable alteration in the levels of systolic blood pressure as illustrated in Figure 11. All the treatment formulations revealed the initiation of their pharmacodynamic effects in varying the systolic blood pressure levels with a statistically significant difference observed among the total duration of treatment period 28th day (*p* <0.05).

It was observed that TTG decline the serum CH level more significantly as compared to PTG (*p* < 0.001), MTG (*p* <0.01) and RTG (*p* <0.05) in comparison to control group.

These data suggested that the drug was more efficient when administered as SNEDDS. These findings proved that SNEDDS can better maintain the potential of Candesartan at an equivalent dose to that of the standard drug solution and marketed tablet. Test formulation has an appreciable effect on the systolic blood pressure profiles of experimental animals in comparison to reference and marketed formulation. Thus, test formulation confirmed extensively better in vivo performance than reference formulation in terms of pharmacodynamic parameters.

## 4. Conclusion

The novel approach was developed for SNEDDS by selecting the optimum concentration of ingredients using a systematic ‘‘DoE’’ methodology of D-optimal mixture design. It has been reported that SNEDDS formulation had a quicker dissolution rate w.r.t. pure drug and marketed tablets which could be attributed to nano globule size and negative value of zeta potential for SNEDDS, which in turn provide greater surface area for the discharge of medicament. The optimized SNEDDS had minimal globule size with the highest rate of drug release. There was no significant difference in the level of Candesartan solubilization under fed and fasting conditions which depicted that SNEDDS can eradicate the influence of food on drug solubilization in vitro. The present investigation has also established that SNEDDS principle is also effective in rats; a significantly improved pharmacodynamic was found when dosed in a SNEDDS compared to a pure drug and marketed tablet with the same dose of drug. Hence, this approach established a considerable improvement in the oral bioavailability of highly lipophilic drugs through the use of SNEDDS. The present study entails the potential effectiveness of SNEDDS with improved release profile and avoidance of food effects and improved pharmacodynamic of medicament w.r.t. pure drug and marketed tablet. The results obtained from a strong rationale for further preclinical studies indicates the potential of SNEDDS as an alternative to oral delivery of Candesartan with enhanced bioavailability and patient compliance and minimal side effects.

## Data Availability

The author authenticates that the data supporting the results and findings of this study are existing within the article.
